# Deciphering melanophagy: role of the PTK2-ITCH-MLANA-OPTN cascade on melanophagy in melanocytes

**DOI:** 10.1080/15548627.2024.2421695

**Published:** 2024-10-30

**Authors:** Na Yeon Park, Doo Sin Jo, Hyun Jun Park, Ji-Eun Bae, Yong Hwan Kim, Joon Bum Kim, Ha Jung Lee, Sung Hyun Kim, Hyunjung Choi, Hyun-Shik Lee, Tamotsu Yoshimori, Dong-Seok Lee, Jin-A Lee, Pansoo Kim, Dong-Hyung Cho

**Affiliations:** a School of Life Sciences, BK21 FOUR KNU Creative BioResearch Group, Kyungpook National University, Daegu, Republic of Korea; b ORGASIS Corp, Suwon, Gyeonggi-do, Republic of Korea; c KNU G-LAMP Project Group, KNU Institute of Basic Sciences, Kyungpook National University, Daegu, Republic of Korea; d R&D Unit, AmorePacific Corporation, Yongin, Gyeonggi-Do, Republic of Korea; e Department of Genetics, Graduate School of Medicine, Osaka University, Suita, Osaka, Japan; f Organelle Institute, KNU, Daegu, Republic of Korea; g Department of Biological Sciences and Biotechnology, College of Life Sciences and Nanotechnology, Hannam University, Daejeon, Republic of Korea

**Keywords:** ITCH, melanophagy, melanosome, MLANA, OPTN, PTK2

## Abstract

Melanosomes play a pivotal role in skin color and photoprotection. In contrast to the well-elucidated pathway of melanosome biogenesis, the process of melanosome degradation, referred to as melanophagy, is largely unexplored. Previously, we discovered that 3,4,5-trimethoxycinnamate thymol ester (TCTE) effectively inhibits skin pigmentation by activating melanophagy. In this study, we discovered a new regulatory signaling cascade that controls melanophagy in TCTE-treated melanocytes. ITCH (itchy E3 ubiquitin protein ligase) facilitates ubiquitination of the melanosome membrane protein MLANA (melan-A) during TCTE-induced melanophagy. This ubiquitinated MLANA is then recognized by an autophagy receptor protein, OPTN (optineurin). Additionally, a phospho-kinase antibody array revealed that TCTE activates PTK2 (protein tyrosine kinase 2), which phosphorylates ITCH, enhancing the ubiquitination of MLANA. Furthermore, inhibition of either PTK2 or ITCH disrupts the ubiquitination of MLANA and the MLANA-OPTN interaction in TCTE-treated cells. Taken together, our findings highlight the critical role of the PTK2-ITCH-MLANA-OPTN cascade in orchestrating melanophagy progression.

**Abbreviations**: α-MSH: alpha-melanocyte-stimulating hormone; dichlone: 2,3-dichloro-1,4-naphthoquinone; ITCH: itchy E3 ubiquitin protein ligase; MITF: melanocyte inducing transcription factor; MLANA: melan-A; NBR1: NBR1 autophagy cargo receptor; OPTN: optineurin; PINK1: PTEN induced kinase 1; PTK2: protein tyrosine kinase 2; SQSTM1/p62: sequestosome 1; TCTE: 3,4,5-trimethoxycinnamate thymol ester; TPC2: two pore segment channel 2; VDAC1: voltage dependent anion channel 1.

## Introduction

Cellular organelles are intricate entities within cells that perform specific functions that are critical for cellular function and survival. These highly specialized structures are subject to precise modulation, undergoing meticulous processes of biogenesis and degradation in response to environmental and functional needs, ensuring the equilibrium and adaptability of cellular life [1,2]. Melanosomes are pivotal cellular organelles in this complex biological system that serve as designers of color and photoprotection in the skin [3]. They facilitate numerous cellular procedures, one of the most important being melanogenesis – a highly regulated, multifaceted process that involves the synthesis, translocation, and release of melanin, the pigment responsible for the color of skin, hair, and eyes [4,5]. α-MSH (alpha-melanocyte-stimulating hormone) is important in stimulating melanin production. When α-MSH binds to its receptor, MC1R (melanocortin 1 receptor), on the surface of melanocytes, it activates intracellular signaling pathways that lead to the production and release of melanin [6–8]. Melanogenesis is predominantly controlled by MITF (melanocyte inducing transcription factor). MITF carefully regulates the expression of several proteins, including TYR (tyrosinase), TYRP1 (tyrosinase related protein 1) and DCT/TYRP2 (dopachrome tautomerase), which are essential for melanin synthesis [9,10]. The modulation of melanogenesis is crucial as it has therapeutic potential, especially in designing interventions for skin hyperpigmentation by targeting the enzymatic activity of melanogenic proteins [5].

Macroautophagy, known as autophagy, is a conserved, lysosome-dependent degradation pathway that is essential cellular homeostasis [11]. Autophagy is characterized by the selective degradation of cellular components, with specific cargo being marked by ubiquitin, regulated by E3 ligases, and subsequently recognized by autophagy receptor proteins such as SQSTM1/p62 (sequestosome 1) [12,13]. Among the various types of selective autophagy, mitophagy is a specialized branch of autophagy that focuses on mitochondrial degradation by autophagy and holds significant prominence [14]. Mitophagy is a well-orchestrated process that begins with the accumulation of PINK1 (PTEN induced kinase 1) on damaged mitochondria, which recruits the E3 ligase PRKN/Parkin. This PINK1-PRKN axis is crucial in mediating the ubiquitination of mitochondrial outer membrane proteins, including the VDAC1 (voltage dependent anion channel 1) and MFN (mitofusin) [15–19]. The ubiquitinated mitochondrial outer membrane proteins are then recognized by autophagy receptor proteins, such as SQSTM1 and OPTN (optineurin), causing the recruitment of autophagic machinery to degrade the damaged mitochondria [17,20]. The integrity of the scenario of mitophagy pathway, PINK1-PRKN-VDAC1-SQSTM1, is critical for maintaining cellular health and preventing the accumulation of dysfunctional mitochondria.

While melanogenesis is critical for the maintenance of melanosome homeostasis by modulating synthesis of melanin, melanophagy, a specialized form of autophagy for melanosomes is important for its degradation [21,22]. This unique interplay between melanogenesis and melanophagy is central for maintaining the balance of melanin within the cell, and it represents a fine interconnection between the production and degradation pathways that determine cellular melanin levels [23]. Recent studies have found a link between autophagy and melanogenesis in melanocytes and keratinocytes, highlighting the concept of melanophagy [24,25]. Notably, 3,4,5-trimethoxycinnamate thymol ester (TCTE, also known as Melasolv™) serves as a potent inhibitor of skin pigmentation by downregulating key pigment-cell-specific enzymes such as TYR, TYRP1, DCT and MITF, pivotal in melanogenesis [26–28]. Intriguingly, our group has demonstrated that TCTE treatment reduces melanin contents by enhancing autophagy [29]. This convergence is critical to understanding skin color variations because the role of autophagy in melanin degradation has significant implications for skin pigmentation [30].

In this study, we elucidated the intricate mechanisms and subtle nuances of melanophagy, using an advanced monitoring system specifically designed for melanosome selective autophagy. Similar to the regulation in mitophagy, we have identified a novel regulatory signaling cascade; PTK2 (protein tyrosine kinase 2)-ITCH (itchy E3 ubiquitin protein ligase)-MLANA (melan-A)-OPTN (optineurin) that controls melanophagy in TCTE-treated cells. Our study of the regulatory dynamics of melanophagy within B16F1 cells aims to improve our understanding of melanin-related disorders and pave the way for the development of innovative cosmetic strategies to address skin pigmentation concerns.

## Results

### ITCH mediates TCTE-induced melanophagy in B16F1 cells

**Figure 1.**
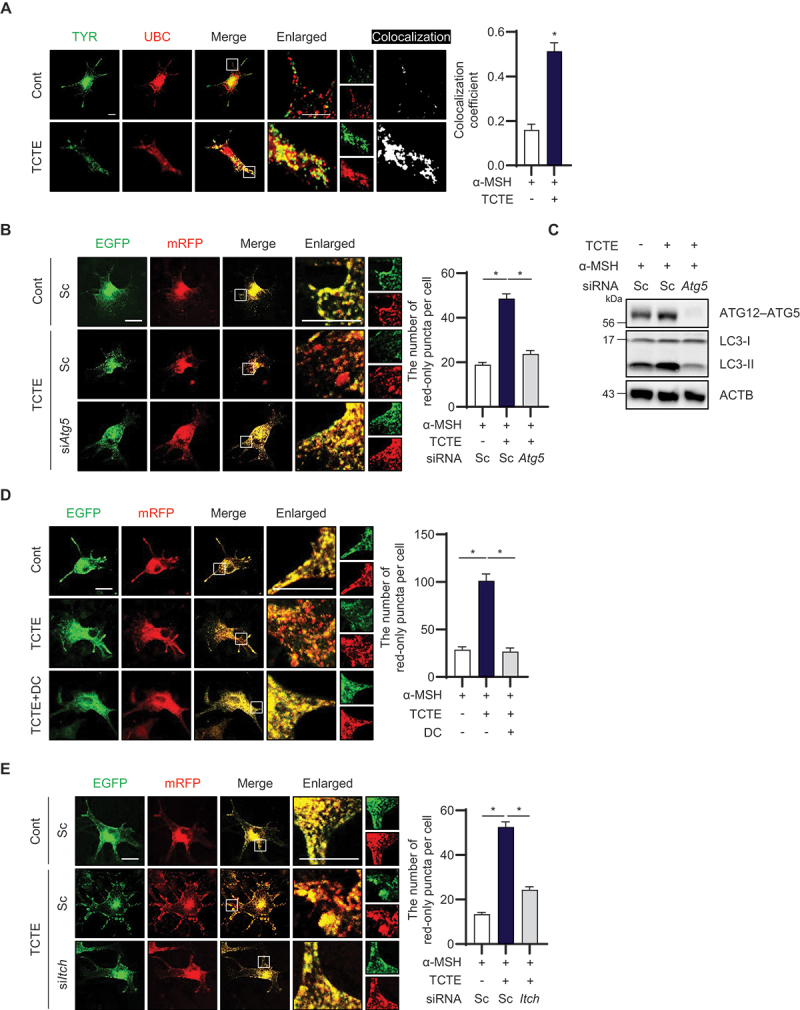
ITCH is involved in TCTE-induced melanophagy in B16F1 cells. (A) α-MSH-stimulated B16F1 cells were transfected with EGFP-TYR and mRFP-UBC. The cells were cotreated with α-MSH (1 μM) and TCTE (10 μg/ml) for 24 h. Then, the cells were fixed and imaged using confocal microscopy. The Pearson’s correlation coefficient was used to calculate the colocalization of TYR with UBC. The data are presented as mean ± SEM (*n* = 6, *p < 0.001). Scale bar: 10 μm. (B and C) B16F1 cells stably expressing TPC2-mRFP-EGFP (B16F1/TPC2-mRFP-EGFP) were transfected with scrambled siRNA (sc) or *Atg5*-targeting siRNA (si*Atg5*). After 1 day, the cells were further treated with α-MSH (1 μM) for 48 h and exposed to TCTE (10 μg/ml) for an additional 24 h. Then, the cells were fixed and the number of red-only puncta per cell was counted using merged images. The data are presented as the mean ± SEM (*n* = 50, *p < 0.001). Scale bar: 10 μm. (C) The knockdown efficiency was assessed by western blotting using indicated antibodies. (D) α-MSH-stimulated B16F1/TPC2-mRFP-EGFP cells were treated with TCTE (10 μg/ml) in the presence or absence of dichlone (DC, 25 μM) for 24 h. Then, the cells were fixed and imaged by confocal microscopy. The number of red-only puncta per cell was counted with merged images. The data are presented as mean ± SEM (*n* = 15, *p < 0.001). Scale bar: 10 μm. (E) B16F1/TPC2-mRFP-EGFP cells were transfected with scrambled siRNA (sc) or *Itch*-targeting siRNA (si*Itch*). After 1 day, the cells were further treated with α-MSH (1 μM) for 48 h and exposed to TCTE (10 μg/ml) for additional 24 h. Then, the cells were fixed and the number of red-only puncta was counted with the merged images. The data are presented as mean ± SEM (*n* = 50, *p < 0.001). Scale bar: 10 μm.

We recently showed TCTE as a strong inducer of melanophagy in hyperpigmented cells [29]. However, the molecular mechanisms underlying melanophagy are largely unknown. In this study, we further explored the mechanisms of melanophagy. Because ubiquitination of target organelles serves as a tagging process marking them for organelle selective degradation, we first investigated whether melanosomes are ubiquitinated during melanophagy. As shown in Figure 1A, TCTE treatment significantly increased the colocalization coefficient between tyrosinase and ubiquitin in α-MSH-stimulated B16F1 cells. Additionally, TCTE enhanced the colocalization of TYR and MAP1LC3/LC3, an autophagosome marker (Data not shown). To investigate TCTE-mediated melanophagy in more depth, we used a monitoring system that addressed the TPC2 (two pore segment channel 2) protein followed by tandem fluorescent tags (TPC2-mRFP-EGFP) [29,31,32]. The number of red-only puncta in B16F1/TPC2-mRFP-EGFP cells treated with TCTE increased significantly, indicating a reduced green signal (mRFP^+^ EGFP^−^) in autolysosomes. However, these mRFP-positive signals were significantly suppressed by *Atg5* knockdown, suggesting that TCTE induces melanosome degradation via activation of autophagy in α-MSH-stimulated B16F1 cells (Figure 1B). Furthermore, LC3-II protein levels were elevated in cells cotreated with TCTE and were blocked by the depletion of *Atg5* (Figure 1C).

To further investigate the molecular mechanisms of melanophagy, we screened a ubiquitination compound library in conjunction with TCTE and identified dichlone (2,3-dichloro-1,4-naphthoquinone) as the most potent novel melanophagy modulator (Figure S1A). To confirm these findings, B16F1/TPC2-mRFP-EGFP cells were treated with dichlone in combination with TCTE. As shown in Figure 1D, dichlone treatment highly blocked the RFP-positive signals increased by TCTE in α-MSH-stimulated B16F1 cells. Previously, it was reported that dichlone derivative 1,4-naphthoquinone inhibits the ITCH, a HECT domain E3 ligase [33]. Therefore, we further investigated the role of ITCH in melanophagy. The increased mRFP^+^ EGFP^−^ puncta signal induced by TCTE was decreased by the depletion of *Itch* expression in α-MSH-stimulated B16F1 cells (Figure 1E and S2A). Additionally, the depigmentation effect of TCTE was attenuated by downregulation of *Itch* (Figure S2D). These results collectively indicate that the inhibition of ITCH reduces melanophagy in TCTE-treated cells.

### MLANA ubiquitinated by ITCH interacts with OPTN to induce melanophagy in TCTE-treated cells

**Figure 2.**
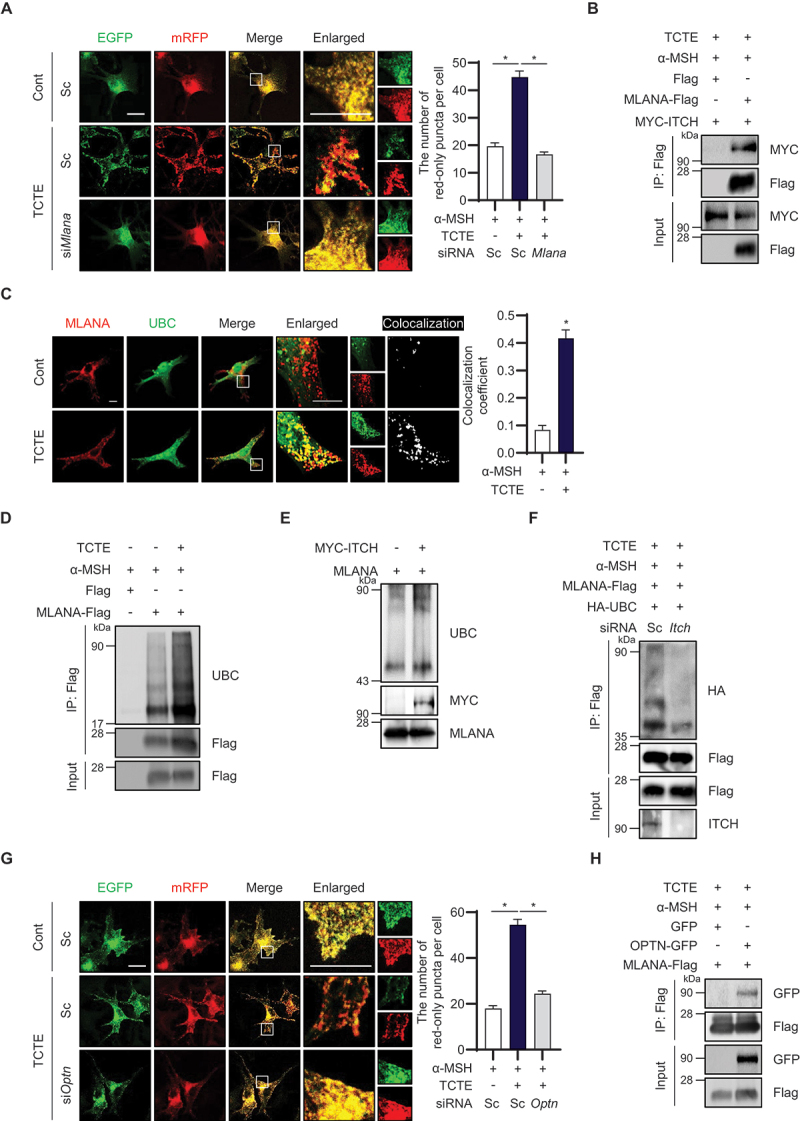
MLANA is ubiquitinated by ITCH and interacts with OPTN in TCTE-induced melanophagy. (A and B) B16F1/TPC2-mRFP-EGFP cells were transfected with scrambled siRNA (sc) or *Mlana*-targeting siRNA (si*Mlana*). After 1 day, the cells were further treated with α-MSH (1 μM) for 48 h and exposed to TCTE (10 μg/ml) for additional 24 h. Then, the cells were fixed and the number of red-only puncta per cell was counted with the merged images. The data are presented as mean ± SEM (*n* = 50, *p < 0.001). Scale bar: 10 μm. (B) α-MSH-stimulated B16F1 cells were transfected with MYC-ITCH in combination with either Flag or MLANA-Flag. After 1 day, the cells were treated with α-MSH (1 μM) for 48 h and cotreated with TCTE (10 μg/ml) with bafilomycin A_1_ (5 nM) for additional 24 h. Then, the cells were further treated with MG132 (10 μM) for 4 h and pulled down with anti-Flag antibody conjugating agarose beads. The immunoprecipitates were analyzed by western blotting with the indicated antibodies. (C) α-MSH-stimulated B16F1 cells were transfected with MLANA-Flag and EGFP-UBC. The cells were cotreated with α-MSH (1 μM) and TCTE (10 μg/ml) for 24 h. Then, the cells were fixed and stained with anti-Flag antibody to get images with confocal microscopy. Pearson’s correlation coefficient was used to estimate the colocalization of Flag with UBC. Data are presented as the mean ± SEM (*n* = 6, * *p* < 0.001). Scale bar: 10 μm. (D) α-MSH-stimulated B16F1 cells were transfected with the Flag or MLANA-Flag for 24 h. Then, the cells were treated with α-MSH (1 μM) for 48 h and exposed to TCTE (10 μg/ml) with bafilomycin A_1_ (5 nM) for additional 24 h. Then, the cells were further treated with MG132 (10 μM) for 4 h and pulled down with anti-Flag antibody conjugated with agarose beads. The immunoprecipitates were analyzed by western blotting with indicated antibodies. (E) *In vitro* ubiquitination assay of MLANA by ITCH. HEK293T cells were transfected with MYC or MYC-ITCH for 24 h. Then, the cells were pulled down with anti-myc antibody conjugating agarose beads. The immunoprecipitates were incubated with purified MLANA proteins for 2 h and then, analyzed by western blotting with indicated antibodies. (F) α-MSH-stimulated B16F1 cells were transfected with scrambled siRNA or *Itch*-targeting siRNA (si*Itch*) in combination with HA-UBC and MLANA-Flag. After 1 day, the cells were treated with α-MSH (1 μM) for 48 h and exposed to TCTE (10 μg/ml) with bafilomycin A_1_ (5 nM) for additional for 24 h. Then, the cells were further treated with MG132 (10 μM) for 4 h and pulled down with anti-Flag antibody conjugated with agarose beads. The immune-complexes were analyzed by western blotting with indicated antibodies. (G) B16F1/TPC2-mRFP-EGFP cells were transfected with scrambled siRNA (sc) or *Optn*-targeting siRNA (si*Optn*). After 1 day, the cells were further treated with α-MSH (1 μM) for 48 h, and exposed to TCTE (10 μg/ml) for additional 24 h. The cells were fixed and the number of red-only puncta per cell was counted with merged images. The data are presented as mean ± SEM (*n* = 50, *p < 0.001). Scale bar: 10 μm. (H) α-MSH-stimulated B16F1 cells were transfected with MLANA-Flag in combination with EGFP or EGFP-OPTN. After 1 day, the cells were treated with α-MSH (1 μM) for 48 h and exposed to TCTE (10 μg/ml) with bafilomycin A_1_ (5 nM) for additional 24 h. Then, the cells were further treated with MG132 (10 μM) for 4 h and pulled down with anti-FLAG antibody conjugated with agarose beads. The immunoprecipitates were analyzed by western blotting with indicated antibodies.

To better elucidate the role of ITCH in melanophagy, we identified potential ubiquitination targets among melanosome membrane proteins in TCTE-treated cells. We created a small siRNA library with various melanosome membrane proteins and collected MLANA as a novel regulator of melanophagy using library screening (Figure S1B, Table S1). To validate these findings, we used B16F1/TPC2-mRFP-EGFP cells, depleted *Mlana* through RNA interference, and treated them with TCTE. As shown in Figure 2A, the downregulation of *Mlana* efficiently suppressed the mRFP^+^ EGFP^−^ puncta signal in TCTE-treated cells after α-MSH-stimulation (Figure 2A and S2B). Nonetheless, the depigmentation activity of TCTE was not significantly influenced by *Mlana* downregulation (Figure S2E).

Then, we examined whether MLANA is substrate of ITCH or not. Importantly, an immunoprecipitation assay revealed that ITCH overexpression is bound to MLANA in α-MSH-stimulated B16F1 cells (Figure 2B). The colocalization coefficient between MLANA and ubiquitin was significantly enhanced in TCTE-treated B16F1 cells (Figure 2C). In addition, ubiquitination of MLANA was highly increased in response to TCTE treatment compared with untreated control cells (Figure 2D). A ubiquitination assay also showed that ubiquitination of purified MLANA protein was increased by ITCH supplement (Figure 2E). Moreover, *Itch* depletion reduced the ubiquitination of MLANA in TCTE-treated B16F1 cells (Figure 2F), suggesting that MLANA is a target for ITCH.

Next, we explored the autophagic receptors implicated in recognizing ubiquitinated MLANA during melanophagy. The downregulation of NBR1 or SQSTM1 did not result in significant change in melanophagy after TCTE treatment in α-MSH-stimulated B16F1 cells (Figure S1C, Table S1). However, the depletion of *Optn* almost completely reversed the increase in red puncta signals, suggesting that OPTN is primarily involved in the recognition of ubiquitinated melanosomes (Figure 2G and S2C). Additionally, the whitening effect of TCTE was reduced by knockdown of *Optn* (Figure S2F). Further immunoprecipitation assays revealed that MLANA interacts with OPTN in TCTE-treated B16F1 cells (Figure 2H). Collectively, these findings indicate that MLANA is ubiquitinated by ITCH and collaborates with OPTN to facilitate melanophagy in TCTE-treated B16F1 cells.

### Activation of PTK2 in response to TCTE phosphorylates ITCH to promote melanophagy in B16F1 cells

**Figure 3.**
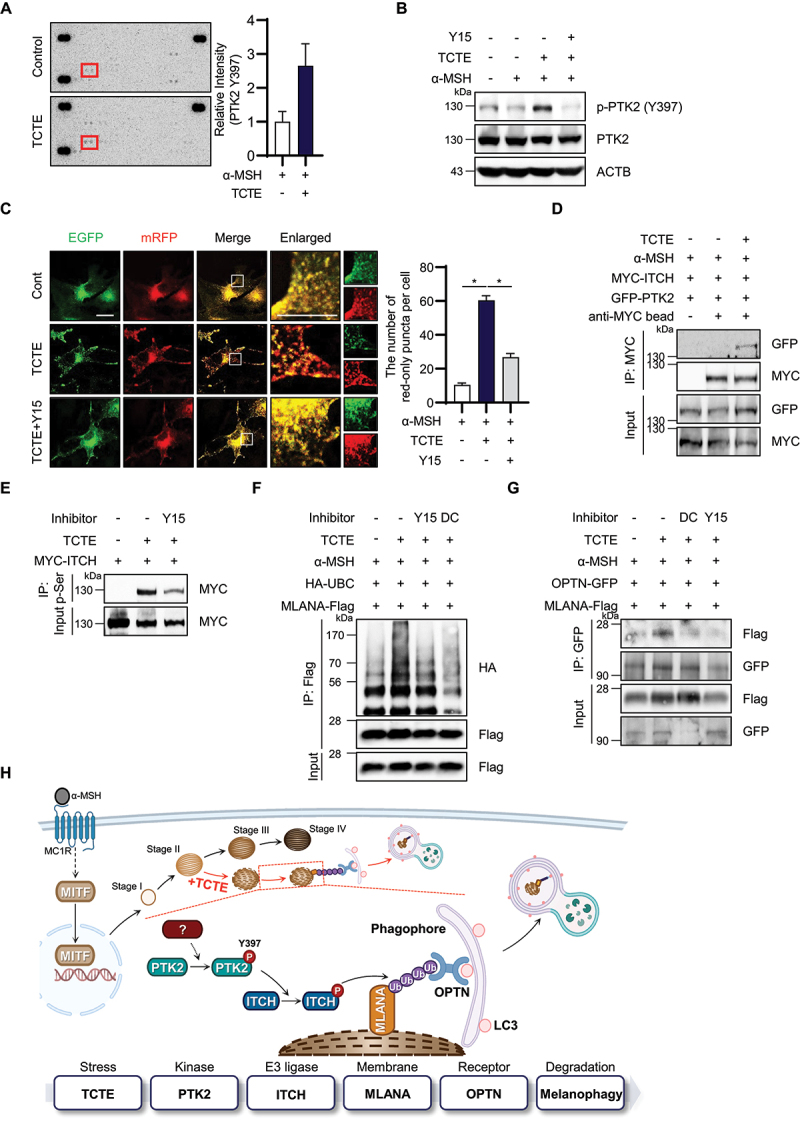
Activation of PTK2 in response to TCTE phosphorylates ITCH to promote melanophagy in B16F1 cells. (A) α-MSH-stimulated B16F1 cells were treated with TCTE (10 μg/ml) for 24 h and subjected to proteome profiler human phosphor-kinase array analysis. Highlighted spots indicate phosphorylated PTK2 at Y397 and the relative intensity was measured by densitometry. (B) α-MSH-stimulated B16F1 cells were cotreated with TCTE (10 μg/ml) and PTK2 inhibitor Y15 (1.5 μM) for 24 h. Then, the cells were harvested and analyzed by western blotting with indicated antibodies. (C) α-MSH-stimulated B16F1/TPC2-mRFP-EGFP cells were cotreated with TCTE (10 μg/ml) and Y15 (1.5 μM) for 24 h. Then, the cells were fixed and imaged by a confocal microscopy. The number of red-only puncta per cell was counted with merged images. The data are presented as mean ± SEM (*n* = 10, *p < 0.001). Scale bar: 10 μm. (D) α-MSH-stimulated B16F1 cells were cotransfected with GFP-PTK2 and MYC-ITCH. After 1 day, the cells were treated with α-MSH (1 μM) for 48 h and exposed to TCTE (10 μg/ml) with bafilomycin A_1_ (5 nM) for additional 24 h. Then, the cells were further treated with MG132 (10 μM) for 4 h and pulled down with anti-MYC antibody conjugated with agarose beads. The immunoprecipitates were analyzed by western blotting with indicated antibodies. (E) α-MSH-stimulated B16F1 cells were transfected with MYC-ITCH for 24 h. The cells were further cotreated α-MSH (1 μM), TCTE (10 μg/ml) and bafilomycin A_1_ (5 nM) for 24 h. Then, the cells were further treated with MG132 (10 μM) for 4 h and pulled down with anti-phosphoserine antibody conjugated with agarose beads. The immunoprecipitates were analyzed by western blotting with indicated antibodies. (F) α-MSH-stimulated B16F1 cells were cotransfected with HA-UBC and MLANA-Flag. After 1 day, the cells were further treated with α-MSH (1 μM) for 48 h and exposed to TCTE (10 μg/ml) with Y15 (1.5 μM) or DC (25 μM) for 24 h. Then, the cells were further treated with MG132 (10 μM) for 4 h and pulled down with anti-Flag antibody conjugated with agarose beads. The immunoprecipitates were analyzed by western blotting with indicated antibodies. (G) α-msh-stimulated B16F1 cells were cotransfected with MLANA-Flag and OPTN-GFP for 24 h. After 1 day, the cells were cotreated with α-MSH (1 μM), TCTE (10 μg/ml) and bafilomycin A_1_ (5 nM) in combination with either dichlone (25 μM, DC) and Y15 (1.5 μM, Y15) for 24 h. Then, the cells were further treated with MG132 (10 μM) for 4 h and pulled down with anti-GFP antibody conjugated agarose beads. The immunoprecipitates were analyzed by western blotting with indicated antibodies. (H) Schematic diagram of a novel melanophagy mechanism involving the PTK2-ITCH-MLANA-OPTN cascade. As a results of TCTE exposure, melanosomes fail to complete maturation. As a sensor of abnormal melanosomes, tyrosine kinase PTK2 is activated by Y397 phosphorylation. Active PTK2 promotes the phosphorylation of the HECT-type family E3 ligase ITCH, and active ITCH promotes the ubiquitination of melanosomal membrane protein MLANA. The ubiquitination of MLANA is recognized by the autophagic receptor OPTN, resulting in melanosomal autophagic degradation.

We further explored the upstream regulatory mechanisms that influence ITCH activity. Analysis using a phospho-kinase antibody array revealed PTK2 was activated after TCTE treatment (Figure 3A). This activation was confirmed by western blot analysis, showing phosphorylation at tyrosine 397 on PTK2 in response to TCTE treatment (Figure 3B). Consequently, we investigated the effect of PTK2 phosphorylation on melanophagy in TCTE-treated B16F1 cells. As shown in Figure 3C, treatment with Y15, a potent and specific chemical inhibitor of PTK2, significantly reduced the mRFP^+^ EGFP^−^ puncta signal in TCTE-treated cells (Figure 3C). Additionally, we discovered that PTK2 aligns with ITCH in response to TCTE stimuli (Figure 3D), implying that the activation is important in TCTE-mediated melanophagy.

Subsequently, we investigated the upstream regulatory mechanisms controlling ITCH activity, and found that ITCH is phosphorylated upon TCTE treatment, a process highly attenuated by the co-application of the PTK2 inhibitor Y15 (Figure 3E). Given the important role of PTK2 in mediating ITCH phosphorylation after TCTE treatment – facilitating the interaction and subsequent ubiquitination of MLANA and the recruitment of the autophagic receptor OPTN – we examined the role of PTK2 and ITCH in ubiquitination of MLANA and recruitment of OPTN. Noteworthily, we discovered that the inhibiting either PTK2 or ITCH with Y15 and dichlone reduces the augmented ubiquitination of MLANA in TCTE-treated cells (Figure 3F). Consistently, an immunoprecipitation assay revealed that Y15 or dichlone treatment disrupts the interaction between MLANA and OPTN, which is enhanced by TCTE treatment in α-MSH-stimulated B16F1 cells (Figure 3G). Collectively, our findings primarily highlight critical role of the PTK2-ITCH-MLANA-OPTN signaling cascade in the orchestration of melanophagy (Figure 3H).

## Discussion

Melanophagy regulates melanin levels by degrading melanosomes, crucial for skin pigmentation and UV protection. Its imbalance of biogenesis and degradation of melanosome can result in pigmentary disorders, emphasizing its significance in skin health [23,30,34]. Previously, we discovered that TCTE promotes depigmentation by increasing autophagic degradation of intracellular melanosomes [29]. However, the precise regulatory mechanisms underlying melanophagy are largely unknown. The present study provides comprehensive insights into the molecular complexities of melanophagy. Of the selective autophagy processes, mitophagy is the most well-defined. Mitophagy involves PINK1 kinase detecting and the E3 ligase PRKN tagging damaged mitochondria by ubiquitinating mitochondrial outer membrane proteins, which are then recognized by autophagy receptor proteins to recruit autophagosomes [35]. According to this mitophagy regulatory scenario (PINK1-PRKN-VDAC1-SQSTM1), our findings suggest the PTK2-ITCH-MLANA-OPTN cascade plays an important role in regulating melanophagy. This novel cascade not only provides a new pathway for understanding melanosome degradation but also provides potential therapeutic targets for conditions associated with altered skin pigmentation.

In this study, we observed that the initiation of the melanophagy pathway is associated with the activation of PTK2, as shown in Figure 3. Although more research is needed to confirm the specific role of PTK2 in melanophagy, its interaction with PPARG/PPARγ (peroxisome proliferator activated receptor gamma) appears significant. Recent studies indicate that TCTE may act as a PPARG agonist, which is supported by the reduced depigmentation observed when using a PPARG antagonist [36]. Furthermore, the decrease in PTK2 phosphorylation in the presence of a PPARG antagonist suggests a possible PPARG-mediated activation of PTK2 [36,37]. Therefore, further investigation is necessary to elucidate the mechanisms underlying this interaction.

Although we could not suggest the target residence of ITCH by PTK2, the phosphorylation of ITCH by PTK2 appears to be a critical event, facilitating the subsequent ubiquitination of MLANA. This ubiquitination is required for melanosomes to be targeted for autophagic degradation. The interaction between MLANA and OPTN, following ubiquitination by ITCH, represents a critical juncture in the recruitment of melanosomes to the autophagic machinery (Figure 3). This process is further supported by the observation that ITCH deficiency suppresses melanophagy, emphasizing the role of ITCH in this pathway. Furthermore, our findings extend beyond cancer cell lines to include non-cancer cell lines such as Melan-A cells, providing additional validation of the melanophagy cascade (Figure S3). Therefore, our discovery enriches our understanding of the regulation of ITCH and highlights the complexity of the signaling pathways involved in melanophagy.

The convergence of autophagy and melanogenesis is not only critical for understanding the physiological processes governing skin pigmentation but also has significant implications for pathological conditions [23,30]. Hyperpigmentation disorders, for instance, might be better understood and managed through targeted interventions that modulate this pathway. The therapeutic potential of manipulating melanophagy is particularly intriguing. By targeting specific components of the PTK2-ITCH-MLANA-OPTN cascade, it may be possible to develop novel strategies for the treatment of pigmentation disorders. For example, the modulation of ITCH activity or the disruption of its interaction with MLANA could serve as a novel approach to control melanin levels in hyperpigmented skin. Our study found that melanin reduction in TCTE-treated cells was reversed by knockdown of *Itch* or *Optn*, but not *Mlana* (Figure S2D, E, F), likely due to the role of MLANA in regulating melanosomal matrix protein, premlanosomal protein for melanosome maturation [38,39]. Further research is needed to understand the precise mechanisms of MLANA in melanosome biogenesis. Furthermore, inhibitors of PTK2 may provide another avenue for regulating melanophagy and, by extension, skin pigmentation. However, the specific effects of TCTE on melanophagy, while elucidated in this study, may differ from those of other compounds or under different physiological conditions. Thus, future studies should aim to validate these findings *in vivo* and explore the potential side effects and efficacy of targeting this pathway in clinical settings.

In conclusion, our findings identified a critical melanophagy regulatory cascade – PTK2-ITCH-MLANA-OPTN that sheds light on melanosome degradation and suggests new treatments for skin pigmentation disorders.

## Materials and methods

### Reagents

TCTE (3,4,5-trimethoxycinnamate thymol ester) was synthesized by Amorepacific Research Group, as described previously [26]. MG132 (474790), bafilomycin A_1_ (B1793) and dichlone (CRM04144) were purchased from Sigma-Aldrich. Y15 (HY-12444) was obtained from MedChemExpress. The short interfering RNA (siRNA) targeting *Atg5* (5´-ACCGGAAACUCAUGGAAUA-3´), *Itch* (5´-GACCUGAGAAGACGUUUGU-3´), *Mlana* (5´-GCUCUGCUUAUCGGCUGC-3´), *Optn* (5´-AGAGAGUGUUGGAAGCGAAGU-3´), and negative scrambled siRNA (5´-CCUACGCCACCAAUUUCGU-3´) were synthesized by Genolution.

### Plasmids

To generate pcDNA/*TPC2*-mRFP-EGFP plasmid, the PCR-amplified products *TPC2* and mRFP-EGFP were individually subcloned into pcDNA3.1/MYC-His (-) A (Invitrogen, V80020). The expression plasmids pCINeo-MYC-ITCH, pOPTN-EGFP, pEGFP-TYR, mRFP-UBC, pEGFP-UBC, pRK5-HA-UBC-WT, pmRFP-LC3 and pGFP-PTK2 were purchased from Addgene (11427, 27052, 32781, 11935, 11928, 17608, 21075 and 50,515; deposited by Dr. Allan Weissman, Beatrice Yue, Ruth Halaban, Nico Dantuma, Ted Dawson, and Kenneth Yamada, respectively). pCMV3-MLANA-Flag (HG-12253-CF) was obtained from Sino biological.

### Cell culture and establishment of stable cell lines

B16F1 cells were obtained from the American Type Culture Collection (CRL-6323). All cells were cultured at 37°C in a 5% CO_2_ incubator and maintained in DMEM containing 10% FBS and 1% penicillin-streptomycin (WELGENE, LM001–05, S001–07 and LS202–02).

To generate stable cell lines, B16F1 melanoma cells were transfected with pcDNA/TPC2-mRFP-EGFP (B16F1/*TPC2*-mRFP-EGFP) using Lipofectamine 2000 (Invitrogen 11,668,019) according to the manufacturer’s protocol. Stable transfectants were selected in a selection medium containing 1.25 mg/ml of G418 (Invitrogen 11,811,023) for 10 days. After seeding the individual cells, the stable clones were selected using a fluorescence microscope (Olympus, IX70, Tokyo, Japan).

### Western blotting

All lysates were prepared with 2× Laemmli sample buffer (Bio-Rad 1,610,737). The total protein was quantified using a Bradford solution (Bio-Rad 5,000,001) as directed by the manufacture’s instruction. The samples were then separated using SDS-PAGE and transferred to a PVDF membrane (Bio-Rad 1,620,177). After blocking with 4% skim milk (MBcell, MB-S1667) in TBST (25 mm Tris-base, 140 mm NaCl [GenDEPOT, T9200 and G0610], and 0.05% Tween® 20 [Sigma-Aldrich, P7949]), the membrane was incubated with indicated primary antibodies: anti-LC3 (NB100–2220; 1:3000) and anti-ATG5 (NB110–53818; 1:3000) antibodies, purchased from NOVUS biologicals; anti-ITCH (611198; 1:1000), obtained from BD Biosciences; anti-MYC (SC-40; 1:1000), anti-GFP (SC-9996; 1:1000), anti-HA (SC-7392; 1:1000), anti-ubiquitin (SC-8017; 1:1000), anti-MLANA (SC-20032; 1:3000), purchased from Santa Cruz Biotechnology; anti-DYDDDDK (2368; 1:3000), anti-PTK2 (3285; 1:1000), anti-phospho-PTK2 Tyr397 (3283; 1:1000), obtained from Cell Signaling Technology; anti-OPTN (ab23666; 1:1000), purchased from Abcam; anti-RFP (MA5–15257, 1:1000), purchased from Invitrogen; anti-ACTB/β-Actin (660009–1-Ig; 1:10000) were purchased from Proteintech. The membranes were incubated with HRP-conjugated secondary antibodies (Cell Signaling Technology, 7076 and 7074; 1:5000). The signals were detected with a Luminograph II (ATTO, Tokyo, Japan).

### Immunoprecipitation

For the immunoprecipitation assay, cells were homogenized in RIPA buffer (50 mm Tris-HCl, pH 7.5, 150 mm sodium chloride, 0.5% sodium deoxycholate, 1% Triton X-100, 0.1% SDS, and 2 mm EDTA; iNtRON Biotechnology, IBS-BR002) containing protease inhibitors (GenDEPOT, P3100–005) at 4°C overnight. For the exogenous IP assay, the supernatant was immunoprecipitated with the following antibodies; anti-Flag-agarose antibody (Abcam, ab1240), anti-MYC-agarose (Santa Cruz Biotechnology, SC-40 AC) and monoclonal anti-phosphoserine-agarose antibody (Sigma-Aldrich, A8076). Following an overnight incubation, the samples were washed twice with RIPA buffer at 4°C and before being added 2× Laemmli sample buffer (Bio-Rad, 161–0737). All samples were analyzed using western blotting as described in the western blotting section.

### In vitro ubiquitination assay

In vitro ubiquitination assay was performed with Ubiquitination assay kit (Abcam, ab139467) according to the manufacturer’s instructions. HEK293T cells (ATCC, CRL-3216) were transfected with plasmids encoding MYC or MYC-ITCH. After 24 h, the cells were homogenized and pulled down with anti-MYC-agarose (Santa Cruz Biotechnology, SC-40 AC) via the same methods with immunoprecipitation section. Purified MYC or MYC-ITCH were incubated with reaction solution (0.1 μM E1 [Abcam, ab139467], 6.6 μg UBE2L3/UBCH7 [R&D system, E2-640-100], 1.25 μg MLANA protein [Proteintech, Ag13387], 2.5 μM Ub [Abcam, ab139467], 0.02 U/μl inorganic pyrophosphatase solution [ThermoFisher, EF0221], 1 mm dithiothreitol [ThermoFisher, R0861], 0.1 M Mg-ATP [Abcam, ab139467], 5 mm EDTA [GenDEPOT, P3100–001]) at 37°C for 2 h. After incubation, the reaction was terminated with 2X non-reducing gel loading buffer (Abcam, ab139467), and the ubiquitination was analyzed with western blotting.

### Confocal microscopy

B16F1 cells were washed twice with 37°C phosphate-buffered saline (PBS; WELGENE, LB001–02) and fixed with 4% paraformaldehyde (Biosesang, PC2031–100) for 15 min at room temperature. To permeabilize, cells were incubated with 0.1% Triton X-100 (GENERAY BIOTECH, 0694) for 5 min and blocked with 2% BSA (GenDEPOT, A0100–010) in PBS for 1 h at room temperature. After blocking, the cells were incubated in primary antibody against anti-Flag (Sigma-Aldrich, F1804) overnight at 4°C. Then, the cells were washed three time with PBS and incubated with Alexa Fluor 555 goat anti-mouse IgG antibody (Thermo Fisher, A21422) for 1 h in the dark at room temperature. The cells were washed with PBS three times and the glass coverslips with cells were mounted on glass slides using anti-fade fluorescence mounting medium (Abcam 104,135). The fluorescence images were captured with a confocal laser scanning microscope (Carl Zeiss, LSM800, NY, USA) and processed with the ZEISS Zen Software.

### Determination of melanophagic cells

To quantify cells with melanophagy, B16F1/TPC2-mRFP-EGFP cells were grown on a cover-glass and transfected with siRNA against *Mlana*, *Optn*, and *Itch*. Then the cells were treated with α-MSH (1 μM, 72 h; Sigma-Aldrich, M4135) or cotreated with TCTE (10 μg/ml, 24 h). The cells were then washed with PBS and fixed with 4% paraformaldehyde for 20 min. Then, the fluorescence images were obtained using confocal laser scanning microscope (Carl Zeiss). The number of melanosomes with only the mRFP signal was counted using ImageJ (NIH) with EGFP and mRFP-merged images.

### Proteome profiler human phosphor-kinase array analysis

To perform Proteome Profiler Human Phosphor-Kinase array (R&D systems, ARY003C), α-MSH-stimulated B16F1 cells were treated with TCTE (10 μg/ml) for 24 h. The cells were washed with cold PBS and lysed with cell lysis buffer containing protease and phosphatase inhibitor cocktail for 30 min at 4°C. Then, the phosphorylation antibody arrays were incubated with the supernatants overnight at 4°C. The arrays were washed and incubated with a detection antibody cocktail and HRP-conjugated anti-rabbit IgG for 3 h at RT, respectively. Chemiluminescence signals were detected with a detection buffer using the Luminograph II (ATTO).

### Statistical analysis

Data were collected from at least three independent experiments and presented as means ± SEM. All statistical analyses were performed using the GraphPad Prism 9 software. The results were statistically analyzed using the *t*-test. A value of * *p* < 0.001 was considered significant for data.
